# Repair of Peripheral Nerve Injury Using Hydrogels Based on Self-Assembled Peptides

**DOI:** 10.3390/gels7040152

**Published:** 2021-09-27

**Authors:** Meng Zhang, Lei Li, Heng An, Peixun Zhang, Peilai Liu

**Affiliations:** 1 Department of Orthopedics and Trauma, Peking University People’s Hospital, Beijing 100044, China; mengzh2008@bjmu.edu.cn; 2 Key Laboratory of Trauma and Neural Regeneration, Peking University, Beijing 100044, China; 3 National Center for Trauma Medicine, Beijing 100044, China; 4 Department of Orthopaedics, Qilu Hospital of Shandong University, Jinan 250012, China; 202036059@mail.sdu.edu.cn; 5 Beijing Key Laboratory for Bioengineering and Sensing Technology, Daxing Research Institute, School of Chemistry & Biological Engineering, University of Science & Technology Beijing, Beijing 100044, China; hengan@xs.ustb.edu.cn

**Keywords:** self-assembled peptides, peripheral nerve injury, design and production process, nerve regeneration, natural polymers

## Abstract

Peripheral nerve injury often occurs in young adults and is characterized by complex regeneration mechanisms, poor prognosis, and slow recovery, which not only creates psychological obstacles for the patients but also causes a significant burden on society, making it a fundamental problem in clinical medicine. Various steps are needed to promote regeneration of the peripheral nerve. As a bioremediation material, self-assembled peptide (SAP) hydrogels have attracted international attention. They can not only be designed with different characteristics but also be applied in the repair of peripheral nerve injury by promoting cell proliferation or drug-loaded sustained release. SAP hydrogels are widely used in tissue engineering and have become the focus of research. They have extensive application prospects and are of great potential biological value. In this paper, the application of SAP hydrogel in peripheral nerve injury repair is reviewed, and the latest progress in peptide composites and fabrication techniques are discussed.

## 1. Introduction

Peripheral nerve injury (PNI) is mainly caused by surgery and trauma, resulting in sensory, motor, and functional disorders of the affected innervated area. Although the peripheral nervous system is more easily regenerated than the central nervous system, the clinical repair of peripheral nerve injury is still not satisfactory [1,2] and remains a great challenge for clinical microsurgery and may also be addressed in terms of basic biology and medicine [3]. Following peripheral nerve injury, a molecular cascade of reactions occurs, involving Schwann cells, fibroblasts, endothelial cells, and macrophages, which are mainly controlled by disintegrating substances and lysing factors of injured nerve axons. This cascade leads to further injury of peripheral nerves [4,5,6]. The in vivo regeneration mechanism is complex, with slow nerve growth, dislocation growth, and target muscle atrophy, resulting in poor functional recovery; only a small number of people are satisfied with their sensory and motor function recovery, and some patients may even be disabled for life [7,8]. This not only creates a psychological obstacle for patients but also a more significant burden to society [9,10]. Therefore, it is necessary to apply various positive methods to promote peripheral nerve regeneration and establish early synaptic connections with target organs to avoid denervated atrophy.

In this paper, the application of different SAP hydrogels in peripheral nerve injury is reviewed, the advantages and disadvantages of various composites are discussed, and possible future directions for the development of nerve repair materials are presented.

## 2. Design of SAPs

Given the developments in microsurgical technology, surgery is the first choice for treating peripheral nerve injury in patients with traumatic peripheral nerve rupture. Together with surgery, the appropriate selection of drugs for adjuvant therapy can improve microcirculation in the injured area, mediate the immune response of inflammatory factors, inhibit oxidative stress reactions, and prevent the formation of nerve scars. In peripheral nerve injury repair research, a combination multiple drugs can achieve a better therapeutic effect than use of a single agent. Still, drug therapy can only be used as a primary treatment for peripheral nerve injury repair, as severe injuries cannot be adequately repaired [11,12].

SAP hydrogels, however, have been applied in the satisfactory repair of nerve injury. Polymeric biomaterials applied after nerve injury mainly play a carrier role, providing a three-dimensional spatial structure for the sustained release of nerve seed cells, growth factors, and drugs that has good biological and cellular compatibility [13]. They, thus, provide a microenvironment for cell differentiation and proliferation, guiding the orderly regeneration of axons in a specific direction, allowing reconstruction of the injured neural network, and promoting functional rehabilitation [14,15,16].

There are also many types of SAPs, of which co-assembling peptides and self-sorting peptides are two that hold great clinical promise. Taking advantage of the co-assembling propensity of self-complementary oligopeptides, Yu et al. co-assembled oppositely charged decapeptides based on either VK or VE to create hydrogel [17,18]. Hahn and his colleagues focus on this co-assembled supramolecular hydrogel and its application in tissue regeneration [19]. Professor Aronsson and others synthesized self-sorting heterodimeric coiled coil peptides hydrogel [20]. This hydrogel shows rapid self-recovery and a satisfactory self-healing capacity.

### 2.1. Commonly Used Repair Sequences

The water content of SAP hydrogels is greater than 99% and they have a good water retention and swelling ratio. This is because SAPs can be assembled and modified in solution to form a hydrogel that has a three-dimensional structure similar to that of human nerve tissue. They can potentially be used as an excellent matrix for the cultivation of nerve cells. In addition, the peptide sequence is easy to design and modify at the molecular level and can be artificially created in advance [21,22]. The methods of synthesis are well developed and easily carried out, and as SAPs are composed of natural amino acids, their degradation products are also small molecular amino acids. In this way, we can avoid the toxic chemical residues and foreign substances in traditional animal-derived tissue materials, which may be related to human immune rejection and cytotoxicity. SAP hydrogels are also beneficial in terms of the myelination of injured peripheral nerve axons.

**Figure 1:**
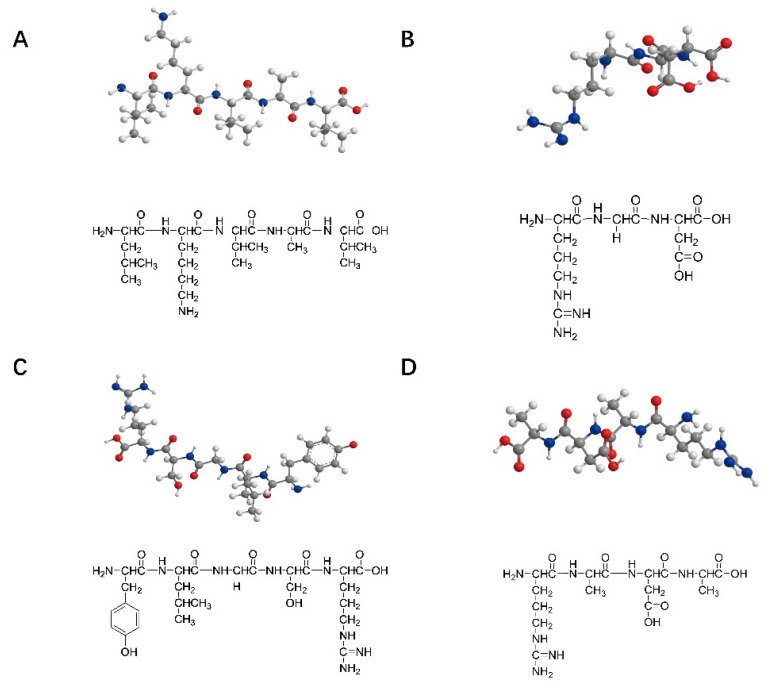
Structures of the SAPs commonly used in nerve repair: (**A**) IKVAV (isoleucine–lysine–valine–alanine–valine); (**B**) RGD (arginine–glycine–aspartic acid); (**C**) YIGSR (tyrosine–isoleucine–glycine–serine–arginine); (**D**) RADA16 (arginine–alanine–aspartic acid–alanine).

The peptide sequences commonly used for nerve injury repair are IKVAV (isoleucine–lysine–valine–alanine–valine), RGD (arginine–glycine–aspartic acid), YIGSR (tyrosine–isoleucine–glycine–serine–arginine), and RADA16 (arginine–alanine–aspartic acid–alanine). These peptide structures are shown in Figure 1 shows. IKVAV can promote the adhesion, differentiation, and axon growth of neurons in the injured peripheral nerve area, inhibit the adhesion and differentiation of glial cells, and simulate the appropriate microenvironment needed for nerve cell growth during the process of peripheral nerve repair [23]. RGD and YIGSR can promote cell adhesion [24,25,26,27]. A self-assembled peptide was designed by Professor Zhai to better control the mechanical strength of the matrix and RGD concentrations, reaching conditions close to those in the extracellular matrix [28]. RADA16, composed of alternating arginine, alanine, and aspartic acid, is a complementary hydrophilic and lipophilic peptide [29]. It can be self-assembled into nanofiber hydrogels with a water content of more than 99% in an aqueous solution that can transport neuro-nutrients to nerve cells and play an essential role in neuronal regeneration, nerve cell adhesion, and axon lengthening. Zhang et al. also successfully designed two functional sequences, IKVAV and RGD-modified RADA16-I SAPs [30,31]. Taiwanese scholars combined the C-terminal of IKVAV peptides and RADA16 polypeptide chains to construct SAPs that can encapsulate neural stem cells in an assembled matrix, thus promoting the regeneration and repair of nerve injury [32].

### 2.2. Composition of SAP Supramolecules

SAP hydrogels can simulate the extracellular microenvironment in the human body and provide good binding sites for cells, being conducive to the adhesion, infiltration, proliferation, migration, differentiation, and synaptic formation of nerve cells. An appropriate spatial structure is necessary such that macromolecules can pass through efficiently and freely [33]. The specific three-dimensional structure depends on the different connection modes between peptides, including hydrogen bonding, electrostatic force hydrophobicity, and π–π interaction. In addition, β-rich structures provided important information for self-assembling peptides [34].

#### 2.2.1. Hydrogen Bonds

During the self-assembly of biomolecule peptides, hydrogen atoms in biomolecules interact with negatively charged particles on the surface of some materials to form hydrogen bonds, which causes the small biomolecules to be arranged in a specific direction in an orderly manner, thus creating a one-dimensional functional nanostructure [35]. At the same time, hydrogen bonding forces, van der Waals forces, and hydrophobic interactions in the peptide α helix play an important role in the overall peptide stability [36]. Hydrogen bonding is essential for the formation of secondary structure in proteins and peptides. The interaction between variable side chains regulates the layout of hydrogen bonds between the leading chains, so there are differences in fiber rigidity among different peptides. Of all the various noncovalent bond interactions of biomolecules, the hydrogen bond may be the most critical link in peptide self-assembly [37,38]. The selectivity of hydrogen bonds induces peptides into a wide variety of three-dimensional nanostructures.

#### 2.2.2. Electrostatic Interactions

Electrostatic interactions play a significant role in peptide self-assembly and mainly exists between the positive and negative ions of peptides. They can be designed into peptides through the addition of small amino acid molecules with positive and negative charges that form ionic bonds as a result of coulombic attraction between the opposite charges, thus self-assembling to form secondary structures and maintain nanostructure stability. The strength of ionic bonds is stronger than those of hydrogen bonds, and electrostatic interactions can be hindered by pH, allowing electrostatic interaction between SAPs to be regulated by adjusting the pH of the assembly solution, thus further affecting its three-dimensional structure.

#### 2.2.3. Hydrophobic Interactions

It is well established that amino acids can be classified as hydrophobic or hydrophilic acids according to the nature of their side chains, and this can determine their agglomeration under certain conditions [39]. Hydrophobic interactions are an important driving force for peptide self-assembly. When amino acid molecules are added to aqueous solutions, the hydrophobic part of the amino acid will orient inward, and the hydrophilic part faces the external solution. Thus, the area of the surface in contact with the aqueous solution is minimized. Although simple hydrophobic interactions are not directional, they can cause peptides or proteins to gather irregularly. Then, under the action of π–π mutual accumulation forces and hydrogen bonding, multiple arrangements will have a clear direction, and they cooperate with each other. Polypeptide self-assembly is enhanced by hydrophobic interactions.

#### 2.2.4. π–π. Interactions

The π–π interactions mainly exist in aromatic amino acids (such as phenylalanine and tryptophan). The benzene rings of aromatic amino acids maintain a vertical and parallel structure that is stable due to the electron cloud attraction between benzene rings [40]. What is more, aromatic interactions can effectively influence the properties of hydrogels. For example, the diphenylalanine hydrogels exhibit higher stiffness due to aromatic stacks [41]. Besides, further addition of konjac glucomannan to the diphenylalanine hydrogel can also control the release rate of the contents [42]. Through π–π interactions, polypeptide fibers can be encouraged to extend in a particular direction. Strongly hydrophobic aromatic amino acids have excellent stability in aqueous solutions. Most peptides will form a hydrophilic and hydrophobic interface with obvious boundaries when interacting with the cell membrane. Therefore, the self-assembly of peptides can be promoted by changing the π–π interaction characteristics of the interface.

## 3. Polypeptide Hydrogel Repairs Peripheral Nerve Injury

A hydrogel is a hydrophilic chain formed by physical and chemical interactions and absorbs water from the surrounding solvent environment during self-assembly; a network polymer with high water content is formed. Hydrogels have good water retention and swelling ratios and a three-dimensional structure, similar to that of human nerve tissue [43]. They can be used as an excellent potential matrix for cultivating nerve cells and can simulate the microenvironment of nerve tissue cytoplasmic matrix [44]. They also allow efficient exchange of substances between cells and their surrounding environment. Polypeptides can be directly injected into injured nerves [45], resulting in spontaneous formation of a hydrogel in the neural cytoplasmic matrix to fill the area where the peripheral nerve is injured and avoid secondary iatrogenic injury caused by a second operation.

Peptides can be injected directly into a site, where they undergo self-assembly into specific functional structures in the humoral environment that are determined by the nature of their predesigned sequences. Specific hydrogel molecular conformations can also be obtained by controlling the pH, light, temperature, and enzyme in advance, which is similar to the extracellular microenvironment in the human body and provides an excellent binding site for cells. This is beneficial to the adhesion, infiltration, proliferation, migration, differentiation, and synaptic formation of nerve cells and inhibits the formation and growth of astrocytes. Thus, due to their capacity for regulation and biocompatibility, peptides have good prospects in the field of peripheral nerve injury recovery [46].

### 3.1. pH Control

**Figure 2:**
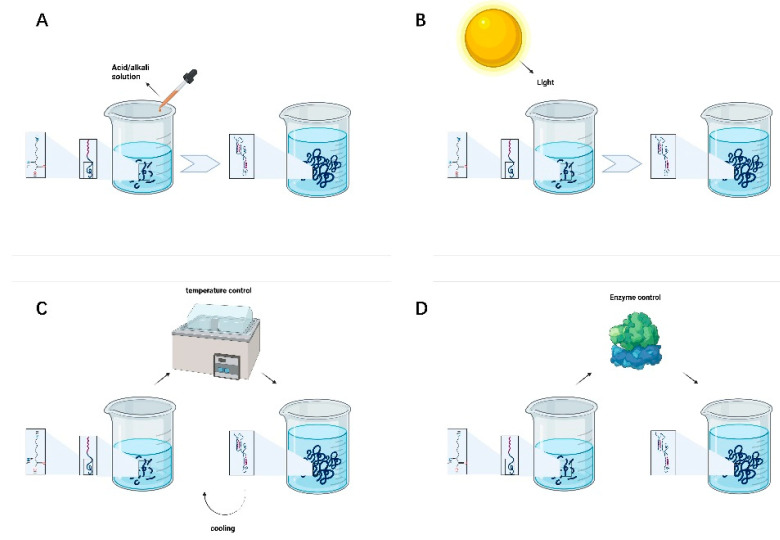
(**A**) pH-controlled peptide self-assembly. (**B**) Light-controlled peptide self-assembly. (**C**) Temperature-controlled peptide self-assembly. (**D**) Enzyme-controlled peptide self-assembly.

Adjusting pH is the most commonly used, convenient, and straightforward method for preparing supramolecular hydrogels. The transition between the hydrogel and solution state of polypeptides can be realized with a change in the solution pH. The pH is particularly important for highly charged amino acid peptide sequences. One of the most critical core driving forces of peptide self-assembly is intermolecular hydrogen bonding interactions. Changing the solution pH of will change the positive and negative charge of the peptide chain, thus resulting in different peptide self-assembly trends and functional characteristics [47]. As shown in Figure 2A, the solution pH can be used to regulate the assembly of SAPs into adaptive hydrogels with different densities and pores to achieve the long-term release of drugs in the area of the peripheral nerve injury and promote nerve proliferation and repair.

### 3.2. Light Control

Photosensitive groups can be introduced into the molecule to control the self-assembly behavior of the polypeptide. The conformational conversion of peptides is not only related to chemical bond breakage s but also the rearrangement of nuclear and electronic structures and can rapidly change under ultraviolet light, visible light, or infrared light irradiation. As shown in Figure 2B, some researchers have demonstrated that different wavelengths of light help predesigned self-assembled peptides to form colloids with various characteristics. The control of peptide self-assembly through a light source has the advantages of high controllability, lack of pollution and direct contact, and reaction accuracy, which significantly facilitates the needs of researchers and patients.

### 3.3. Temperature Control

Temperature will destroy hydrogen bonding and hydrophobic interactions, resulting in changes in the molecular structure. The most commonly used method to control the reversible self-assembly of peptides is temperature. Different temperatures can be used to realize the transformation of polypeptide molecules between a sol and a gel, which is reversible in a specific temperature range; see Figure 2C. A current research hotspot is focused on developing drugs to promote peripheral nerve injury repair by adjusting and controlling the gel-forming temperature of polypeptide hydrogels. By controlling the system temperature to induce drug aggregation or release via changes in the self-assembly state of peptides, the regulation of drug concentration in the peripheral nerve injury area can be improved and controlled, and the therapeutic effect can be enhanced.

### 3.4. Enzyme Control

Enzymes can be employed to specifically degrade required target groups in the peptide chain. They can also catalyze binding of a compound to the peptide, thereby changing its hydrophilicity/hydrophobicity. Phosphokinase and phosphatase can phosphorylate and dephosphorylate the hydroxyl groups of polypeptide chains, respectively, altering hydrogen bonding interactions between polypeptide chains, destroying or restoring order in the self-assembled structure [48]. As shown in Figure 2D, enzymatic reaction can accelerate the self-assembly process. Enzymes can promote the formation of various nanostructures and encourage capillary regeneration, thus nourishing the injured peripheral nerves and tissues.

## 4. Repair Method

### 4.1. Promote Cell Proliferation, Migration, and Reprogramming

**Figure 3:**
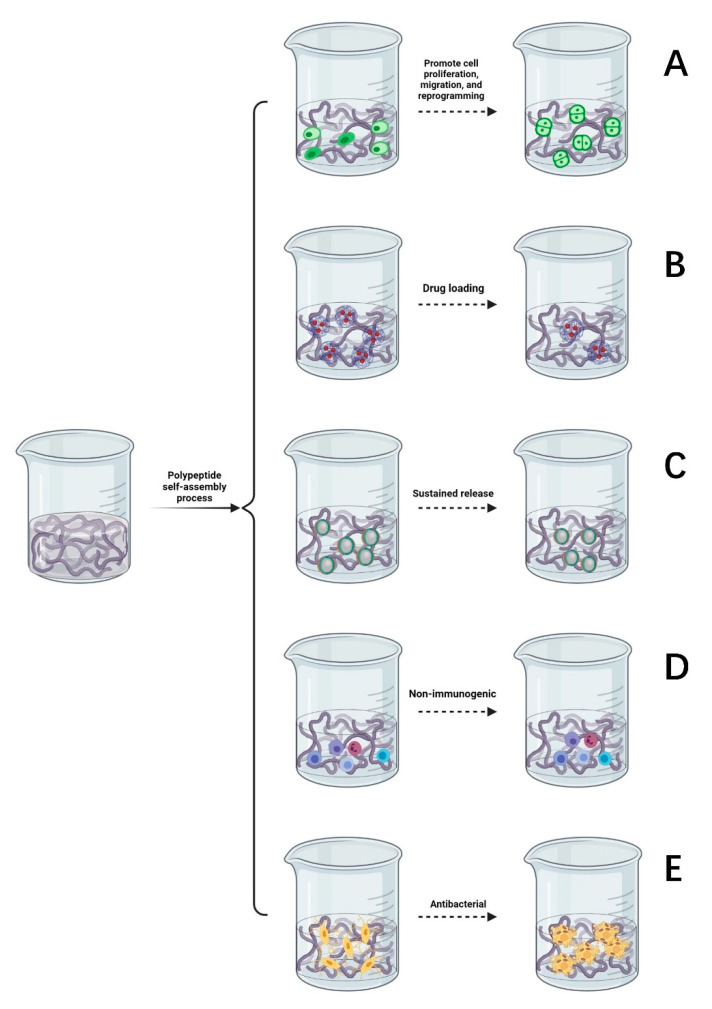
(**A**) Self-assembled peptides promote cell proliferation, migration, and reprogramming. (**B**) Drug loading function of a self-assembled peptide hydrogel. (**C**) The slow-release process of a self-assembled peptide hydrogel. (**D**) The self-assembled peptide matrix is non-immunogenic. (**E**) Antibacterial properties of self-assembled peptide matrix.

Supramolecular hydrogels formed by the self-assembly of peptides are widely used in the culture of nerve tissues and nerve cells, promoting cell proliferation, migration, and reprogramming [49]. SAP hydrogels provide a good environment for regeneration following peripheral nerve injury, prompting nerve cells to migrate, slowly absorb nutrients and growth factors, and expel metabolic waste [50]. They are an ideal culture medium and material to promote the differentiation and growth of nerve cells in regenerative medicine [51,52]. Some studies suggest that SAP hydrogels can promote the directional differentiation of stem cells [53]. As described in Figure 3A, co-culture of self-assembled hydrogel and nerve cells can reduce the incidence of nerve cell apoptosis, promote the survival, growth, and proliferation of new nerve cells, and emit nerve axons in a specific direction to facilitate the formation of active synapses between neurons, so as to promote the functional recovery of the injured area of the peripheral nerve.

### 4.2. Drug Loading

SAP hydrogels can be used as carriers to deliver drugs whereby the high loading of nerve growth drugs can be controlled such that there is a limited dose is released to specific peripheral nerve injury sites. After the drug is released, peptides can be degraded into safe, nontoxic metabolites [54]. It has been found that SAP hydrogels can transport more drugs and plasmid DNA to the target region and upregulate the expression of related genes [55]. As demonstrated in Figure 3B, the drug delivery system of SAP hydrogels will form a protective shell on the surface of hydrophobic compounds, thereby improving permeability. Hence, it is easier for drugs to penetrate the cell membrane instead of through interacting with receptors on the surface and the exposure of drugs to degradants is reduced [56,57]. SAP hydrogels can also prevent contact between cytotoxic drug molecules and parts outside the target tissue and improve the utilization of drug components through increased efficiency [58,59,60]. Based on this exceptional property, some studies have shown that self-assembled peptide carriers have the advantages of reducing drug toxicity, prolonging the drug half-life, and increasing the drug solubility and biodegradability [61,62].

### 4.3. Sustained Release

The variable three-dimensional structure of self-assembled peptide hydrogels means that their structure can be manipulated such that they serve as a sustained release carrier for many types of drugs. The results show that the self-assembling polypeptide RAD-16 can be used as a recombinant adenovirus gene-controlled release vector [63]. Optimization of the gel surface structure provides a space for cell adhesion and delays the release of growth factors [64,65], as described in Figure 3C. The hydrogels formed by the self-assembly of two oppositely charged peptides form a complex with charged drugs, through electrostatic interactions, to form excellent materials for the sustained release of drugs. Amphiphilic peptides can self-assemble with the medications used, forming stable complexes that can reduce drug toxicity, carry them through the nerve cell membrane, and continuously release drugs that nourish nerves, achieving a controllable sustained-release effect. This effectively promotes the directional growth and migration of nerve cells and enhances the therapeutic effect of drugs on peripheral nerve injury.

### 4.4. Nonimmunogenic

As suggested in Figure 3D, self-assembled polypeptides are not immunogenic. Therefore, developing SAPs that present immunogenic antigens has reasonable application prospects [66]. Long, unbranched nanofibers are formed in peptide self-assembly system and can effectively wrap the antigenic epitopes of pre-immunogenic drugs, reduce the level of immunity required by the body while simultaneously reducing the adjuvant effect [67]. In terms of immunology, SAP hydrogels can be used as carriers of antigens and antibodies, genes, and proteins, as well as biological sensors to recognize and regulate gene expression, thus regulating the behavior of stem cells. Compared with ordinary antibodies, SAP hydrogels have such advantages as low production cost, low immunogenicity, good penetration, and a potent anti-inflammatory effect. Based on the above benefits, SAP hydrogels are more often being used to treat peripheral nerve injury and wound repair.

### 4.5. Antibacterial

Due to the massive abuse of antibiotics, the problem of the antibiotic resistance of bacteria has become increasingly prominent. There is an urgent need to develop new antimicrobial agents with low drug resistance. It has been reported that inflammation can aggravate the degree of nerve injury [68]. As shown in Figure 3E, self-assembled antimicrobial peptides have the advantages of a good bacteriostatic effect, high biocompatibility, flexible design, and rich amino acids. Self-assembled antimicrobial peptides can be used to increase the permeability of bacterial cell membranes. Nanotubes formed by hydrogen bonding between peptides are selectively embedded in the lipid bilayer of the bacterial cell membrane, and ions are transported through tubular channels across the membrane. Increasing bacterial membrane permeability can shorten the germicidal time, which can reduce the likelihood of drug resistance developing [69]. After the drug enters the bacteria, the hydrophilic and hydrophobic properties of the SAPs are altered through temperature control or light to form a viscous hydrogel that fixes bacteria and prevents them from migrating. In the area of peripheral nerve injury, SAPs further form nanofibers, and nanonetworks are used to capture the remaining bacteria, preventing them from invading and hindering their proliferation to prevent infection from spreading to other tissues and thus delaying bacterial growth.

## 5. Other Application of Peptide-Based Hydrogels

For decades, polypeptide hydrogels have been used extensively for drug and gene delivery in pharmaceutical applications. For example, poly(glutamic acid) micelles are doped with cisplatin to improve the accumulation of chemotherapeutic agents in tumor tissue and to reduce their side effects [70,71]. The greatest effect in oncology therapy is obtained when self-assembly is induced by covalent coupling with hydrophobic small molecules such as adriamycin and salinomycin [72,73,74,75]. The gel bundles formed are also very effective when coalescing to form drug deposits after injection [76]. Amino acids based as gene delivery systems, such as poly(ornithine) and poly(lysine), have demonstrated the ability to silence gene expression and gene transfection for the treatment of the central nervous system and cancer [77,78,79]. Hydrogel systems based on self-assembled peptides have also been developed in tissue engineering and regenerative medicine [80,81,82]. The application of poly(amino acids) as therapeutic agents has been investigated [83]. Self-assembled peptides based on nanorods can act as immunostimulatory factors and delivery systems comparable in size and shape to a variety of invasive pathogens and display repetitive antigens on their surface [84]. It is a promising platform for delivering self-adjuvanted antigenic epitopes that can completely prevent infection with the deadly H1N1 influenza A virus. This peptide is already evolving as an advanced adjuvant in the preparation of vaccines. In addition, Professor Diaferia et al. have reviewed that polypeptide-based Gd supramolecules can be used as an MRI contrast agent [85]. In the diagnosis of pathology, the fluorescence effect of peptide derivatives can effectively show amyloidosis [86]. Moreover, metal ion-excited peptide gels have better responsiveness and a wider range of clinical applications [87,88,89].

## 6. Conclusions and Future Prospects

The repair of peripheral nerve injury has always been a complex problem in medicine because of its high incidence, high disability rate, low cure rate, and heavy social and personal burden. In only relying on the traditional treatment of peripheral nerve injury, such as surgery or drugs, it is no longer possible to meet the high requirements to achieve a therapeutic effect in modern patients. The research results of scientists in various countries have demonstrated the benefits of SAP hydrogels in repairing peripheral nerve injury. 

In this paper, the strategies commonly used for nerve injury repair are summarized. The molecular bonds and interactions that play an important role in the structural design of SAP hydrogels are systematically explained. Due to the dynamic changes in environmental conditions such as pH, temperature, light intensity, and enzymes, the fact that they can change the structural properties of SAP hydrogels, which are thus controllable, is particularly advantageous for ensuring their wide used. Supramolecular nanofiber hydrogels with extremely high water content can self-assemble into supramolecular nanofiber hydrogels [90]. This kind of hydrogel can be used as a suitable matrix for cultivating nerve cells, which is beneficial to the adhesion, infiltration, proliferation, migration, differentiation, and synaptic formation of nerve cells and fills the injured area of the peripheral nerve without causing cytotoxicity. This is conducive to application in the myelination of injured peripheral nerve axons. Furthermore, SAP hydrogels have excellent biological activity, biodegradability, and biocompatibility. As drug delivery carriers, the specific hydrophobic drugs are delivered to particular peripheral nerve injury sites with high loads. The nutritive nerve drugs are continuously released to achieve a controllable slow-release effect to achieve nerve repair. In the clinic, improving the repair effect of peripheral nerve injury can be achieved through the selection of potential new materials for use in hydrogels, which is a prospect for SAPs. In the future, predesigned peptides with good biocompatibility will have broad application in the fields of nerve repair and biological tissue engineering.

## Data Availability

Not applicable.
